# The quality of life of patients with genital warts: a qualitative study

**DOI:** 10.1186/1471-2458-10-113

**Published:** 2010-03-07

**Authors:** Gitte Lee Mortensen, Helle K Larsen

**Affiliations:** 1 Medical anthropology, AnthroConsult, Fynsgade 24, 8000 Aarhus C, Denmark; 2 Venereal Diseases Clinic, Bispebjerg Hospital, Bispebjerg Bakke 23, 4 Tvaervej, 1 sal, 2400 Copenhagen NV, Denmark

## Abstract

**Background:**

Genital warts, which are caused by infection with human papillomavirus (HPV), are one of the most common sexually transmitted diseases in Europe. Although genital warts are commonly perceived as a non-serious condition, treatment is often long, of varying effectiveness and the recurrence rate is high. Very few studies have been performed on the personal consequences of genital warts. The aim of this qualitative study, set in Denmark, was to examine the ways in which genital warts may affect patients' quality of life.

**Methods:**

To obtain an in-depth understanding of patients' perceptions of genital warts, we used qualitative focus-group interviews with five men and five women aged between 18 and 30 years who had genital warts. The interview guide was based on a literature review that identified important issues and questions. The data were analysed using a medical anthropological approach.

**Results:**

Patients' experiences were related to cultural conceptions of venereal diseases and the respective identities and sexuality of the sexes. The disease had negative psychological and social effects both for men and for women and it affected their sex and love lives, in particular. The psychological burden of the disease was increased by the uncertain timeline and the varying effectiveness of treatment. We identified a need for more patient information about the disease and its psycho-sexual aspects.

**Conclusions:**

The men and women participating in this study considered their quality of life to be significantly lowered because of genital warts. The experiences described by the participants give insights that may be valuable in treatment and counselling.

The quadrivalent HPV vaccine that has now been added to the childhood vaccination programme for girls in Denmark for the prevention of cervical cancer can also prevent 90% of cases of genital warts. Our results suggest that HPV vaccination could considerably reduce the largely unacknowledged psychological and social burden associated with genital warts, in men as well as women.

## Background

It was recently reported that 10.6% of women aged 18-45 years in northern Europe have had genital warts (GWs) and that the incidence of GWs among young women is rising [1]. In the United Kingdom, for example, GWs are now the most prevalent venereal disease and the number of reported cases has increased by almost ten times within the past 30 years [2]. The treatment of GWs is often long and of varying efficacy [3]. In Denmark, a country with 5 million inhabitants, 17% of women aged 20-29 years have had GWs [4]. The Danish National Health Board has estimated that annual expenditure on treatment of GWs is approximately 3.9 million euros [5].

GWs are caused by infection with certain types of human papillomavirus (HPV). More than 100 types of HPV exist, of which between 30 and 40 are associated with the mucosa and skin of the anogenital area [6-8]. Approximately 90% of cases of GWs are due to infection by HPV types 6 and 11. The use of condoms reduces but does not eliminate the risk of HPV infection [9]. Although most HPV-related genital lesions resolve spontaneously within 1-2 years, there is no specific treatment for persistent HPV infection [10]. Most patients require more than one course of treatment for visible GWs, and the choice of therapy depends on the location, number and size of the lesions. Management options include patient application of 0.5% podophyllotoxin, 5% imiquimod cream or 5% 5-fluorouracil cream, or physician application of 20% Podophyllin resin, cryotherapy, surgical excision or carbon-dioxide laser. All treatments are associated with a degree of discomfort for the patient and subsequent local reactions such as burning, irritation of the mucosal membranes and ulceration. The efficacy of the different treatments varies between 20% and 60% and the rate of recurrence of GWs is high [11-15].

Public interest in HPV vaccination has so far centred on the possibility of preventing cervical cancer. Several European countries, Australia and the USA have introduced HPV vaccination programmes for young girls to prevent cervical cancer. In Denmark, for example, HPV vaccination of 12-year old girls was introduced to the children's vaccination programme in the autumn of 2008 [5,16]. However, the quadrivalent HPV vaccine (Gardasil^®^) protects not only against HPV types 16 and 18, which cause approximately 70% of cervical cancers, but also against types 6 and 11 and thus has the potential to prevent 90% of GWs [17,18].

GWs are often perceived as benign and non-serious infections, and there have been few studies on the quality of life of patients with GWs; the majority of studies on HPV-related diseases concern women's experiences with cervical dysplasia and cervical cancer [19-31]. Quality of life is defined here as the psychological, social and physical well-being of the patient. The sparse literature identified during the present study [3,14,15,32-45] indicated that patients with GWs suffer anxiety about the effect of the disease on their love life and sexual [3,32,36-38,40,43,44] and social relationships [32,36], the stigma of having contracted a venereal disease [34,35,38,39,43,45], the uncertain treatment success and time to cure [3,14,32,34-36,40,44] and transmission of the disease to others [3,32,36,40,37]. Several studies report that the negative psychological effects of the disease are the most difficult [14,35,36,38,43,45]. They include feelings of anger, fear caused by the relationship of HPV to cervical cancer, guilt, depression, self-loathing and worries about the future [3,14,32,34-40,42-45]. Finally, the literature points to a huge need for more information about the disease and an improved doctor-patient communication [3,14,15,33,35,36,38,41,43,45]. Of the 17 relevant articles identified [3,14,15,32-45], only two were based on qualitative studies [3,32]. The objective of this qualitative study, the first of its kind in a Danish context, was to gain an in-depth understanding of the ways in which GWs may affect patients' quality of life.

A qualitative approach is the most appropriate to examine patients' perceptions of a disease in a given socio-cultural context. The strength of the qualitative approach lies in its ability to explain patterns of meaning and answer questions such as, 'what?', 'how?' and 'why?', rather than 'how many?' or 'how often?'. The results can be generalised analytically in the sense that we gain knowledge about the qualities of a phenomenon regardless of the frequency of its occurrence [46].

**Figure 1:**
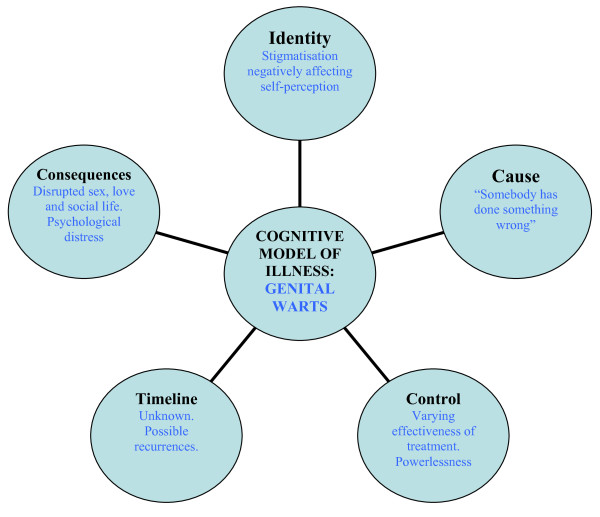
**Application of the cognitive model of illness (comprising five core dimensions) to the case of genital warts**. Modified from [34].

The medical-anthropological basis for this study was that a person's perception of a disease influences his/her personal experience of it. Kleinman has proposed a distinction between the terms *disease*, referring to the biomedical condition from the practitioner's point of view, and *illness*, referring to the patients' perception of the condition and coping with it [47,48]. According to the illness paradigm, patients create cognitive models of their illness which constitute five core dimensions: identity, cause, control, timeline and consequences (Figure 1). The perception of each of these dimensions influences how patients cope with their illness. For instance, the degree of anxiety caused by an illness is greater if the perceived consequences are serious, if the patients feel they have no control over the illness, if the treatment effectiveness is poor or if the time to cure is long and uncertain [34]. In this study, we aimed to examine patients' cognitive models of genital warts.

## Methods

The present study was based on qualitative focus group interviews with both men and women. This methodology, focus groups, was chosen to create a confidential setting in which people could openly and anonymously discuss their experiences of the disease. The aim was to gain an insight into as broad a range of perspectives on the disease as possible. At the same time, using focus groups allowed us to observe the dynamic and social construction of GWs. The groups were small and single-sexed to help the participants to feel more comfortable talking about the subject. We recruited participants aged between 18 and 30 years because the prevalence of GWs is highest in this age group, and also because being of a similar age usually helps to encourage honest and open group discussions [49,50].

The 10 participants (five women and five men) were recruited from the venereal diseases clinic at Bispebjerg Hospital in Copenhagen, Denmark, which has approximately 8000 consultations for GWs per year. Participants were eligible if they were aged between 18 and 30 years old, had suffered from GWs for at least three months, were seeking treatment at the time of inclusion, and did not have any serious co-morbidity or any other sexually transmitted disease. Patients who fulfilled the inclusion criteria were informed orally about the study by the consulting physician, who also gave them a study information sheet. Patients wishing to participate then contacted GLM, who acted as an independent researcher, and they all granted their informed consent. No personal information about the participants was passed on from the venereal diseases clinic to the authors or other people involved in the study. The participants' anonymity was ensured throughout the study that did not require ethics committee approval in Denmark. All the participants were ethnically Danish.

The focus-group discussions were held after working hours in a small library at the Venereal Diseases Clinic, because this was a neutral but familiar environment. The focus groups were moderated by GLM with the assistance of an experienced anthropology student. The purpose and design of the focus group was explained before the interviews started. A semi- and funnel-structured interview guide (Table 1) was used to moderate the focus-group discussions. Following the cognitive illness model, the interview guide began with questions to the participants' perceptions of genital warts: the perceived causes, means of controlling and time-line of GW. Subsequently, more focus was brought on the personal consequences of having GW, including the effects on patients' identity. With this aim, the interview guide covered topics and questions that were identified and formulated on the basis of a literature search using PubMed, Embase, CINAHL and Psycinfo. The questions were open-ended to capture as many perspectives as possible, including any that had not been envisaged by pre-interview hypotheses [49].

**Table 1:** Interview guide

Interview stage	Aim	Question
Opening	Presentation of participants	What is your first name, age and how long have you been suffering from GWs?

Introductory question	The participants' reaction to the diagnosis of GWs	1. How did you react when you heard that you had GWs?

Transitional question	Perception of illness	2. What are your views on this disease (in comparison to other STDs, for instance)? *Cues*: *Cause, consequences, treatment options, time to cure and knowledge about HPV*

Key questions	The effect of GWs on their quality of life	3. How has your lives been affected by having GWs? a. Physical effects b. The course of treatment
		*Cues: Pain, embarrassment, worries about treatment effectiveness and duration of treatment, information and knowledge, doctor-patient communication, practical aspects*
		c. Effects on work and studies
		*Cues: Concerns about stigma, effect on ability to work or study, sick leave, contagion, social avoidance*
		d. Social relations
		*Cues: Concerns about stigma, contagion, avoidance, social isolation*
		e. Love life
		*Cues: worries about present or future partners, infidelity, contagion, conflicts about infidelity or disrupted sex life, fear of rejection and condemnation/disapproval*
		f. Sex life
		*Cues: Desire/lust, initiative, pleasure, spontaneity, avoidance, low self-esteem, negative body-perception, fear of rejection, lack of sexual ability*
		g. Psychological effects
		*Cues: Guilt, shame, anger, worries about the future, depression, fear, negative self-perception, identity, disease phobia*

		4. Has your quality of life changed since you were first diagnosed with GWs, and if yes, how?
Closing questions		5. In conclusion, I would like each of you to tell me which of the areas that we have discussed has been most affected by having GWs? 6. Is there anything you think we ought to have discussed but did not?

GWs, genital warts

The focus-group discussions were transcribed verbatim and analysed using NVivo, a software programme for analysing qualitative data (QSR International). A social constructivist approach to the relationship between language and the social construction of meaning was used to analyse the data [50]. This approach is used to analyse a diversity of statements such that clusters of meaning around specific subject matter are generated. It involves an analysis of the terminology used to speak about the subject and the ways in which it is related to other issues. Firstly, the data were coded into the topics that were brought up during the discussions. Secondly, the most important themes within each topic were identified. Finally, the frequency of and connections between topics and themes were analysed. This generated a pattern of the relative meaning that the different topics and themes had for the participants, i.e. the most significant ways their quality of life had been affected by having GWs. All methodological and analytical steps were discussed with an anthropologist who was not involved in the project.

## Results

The average age of the men and women in this study was 25.8 years and 24.4 years, respectively. The participants had had their present case of GW between 3-38 months at the time of the focus groups (Table 2). This implies an average duration of 11 months. For seven participants this was their first case of GW, while three participants had an earlier (first) case of GW.

**Table 2:** Focus group participants

Women		Men	
**Age** **(years)**	**Duration of present case of GW (months)**	**Age** **(years)**	**Duration of present case of GW (months)**

23	18	22	8 (84)*

24	4	30	3 (10)*

21	8	28	12

25	38	26	8

29	3 (24)*	23	8

*Parentheses referring to number of months since clearing of a first case of case of GW, if any.

The participants were extremely frank during the focus groups and several said that they had been glad to be able to speak openly (for many, for the first time) with like-minded people about their illness. Many participants said they had volunteered because they wanted to participate in the generation of knowledge about GWs and its personal consequences because they felt that the disease was ignored, compared with other venereal diseases.

The quotes cited below were selected because they illustrate some of the participants' most important experiences with GWs.

### Illness perception: the cognitive model of genital warts

The participants regarded GWs as a stigmatising venereal disease. This was expressed as shame and feelings of being impure and repulsive. At the time of diagnosis, most participants did not know that the virus can be carried for some time before the GWs develop. Uncertainties about the source of the infection often led to worries about infidelity within the patients' relationships. The causal explanation of the illness was closely associated with the idea that "somebody had done something wrong".

The participants' views on having GWs had changed since the time of diagnosis. The majority did not know much about the disease before and had initially been optimistic about it being cured. Pessimism had gradually set in as it became clear that treatment can be complicated. The long and uncertain timeline as well as the psycho-sexual consequences of the disease had taken the participants by surprise and this had increased the burden associated with GWs over time.

I just went "s**t" but then I thought "well, it'll soon pass. I'll just get a pill or something [she laughed]. But it wasn't exactly like that. Now, I've changed my perception pretty much. I'm really sick of it now. [23-year-old woman]

A few participants regarded GWs as a very serious condition because they thought that GWs increased the risk of developing cervical cancer. The majority, however, considered that GWs had mainly important psycho-sexual effects on their quality of life.

### The effect of genital warts on the patients' love and sex lives

The majority of participants indicated that it was their sex and love life that had suffered most from having GWs. Their libido was low and their sexual initiative was reduced, and pleasure and spontaneity was often lost during intercourse because of awareness of the warts, fear of transmitting the disease or repulsing the partner, negative self-perception and soreness due to treatment. This affected steady relationships and for those who were single it affected their ambition to seek a new partner.

*It [the GW] has definitely had a huge impact on my sex life. It's a barrier for meeting new girls. 'Cause I stand there thinking "Wow, she's nice. I'd like to take her home". But then, I don't want to approach her 'cause... I'm simply not up to explaining it. To me, it has meant that I don't really feel like going out and looking for a new steady partner. That also totally destroys your self-confidence, I mean, that you actually lose the desire to meet girls*. [26-year-old man]

The participants described themselves as 'impure, repulsive and sexually unattractive' and seriously questioned how others might find them attractive when they did not even like themselves. Problems often arose in existing relationships because of worries about the source of the infection or a lack of sexual desire. Men, in particular, often felt a pressure for sexual affirmation.

*I think they [the GWs] affect you a lot, especially when you kind of lose the desire for sex. I mean, it's not that you don't think your girlfriend's attractive or anything... It's like, it affects you psychologically and you say: "No, I don't think so. I don't really care that much for myself when I'm like this". This resulted in my girlfriend feeling that I didn't find her sexy or beautiful, and just saying that she is wasn't enough all of a sudden. And you can't really do much more, 'cause if you don't feel like having sex, well, you just don't feel like it, damn it! [...] One of the most tedious aspects of being treated with cryotherapy is that nothing bloody works until the ulcers heal again - and then you have to almost train it again afterwards. It's also really stressful in that when it finally works again, you feel that you have to perform because the tool has been out of order for a while. At least, until my girlfriend and I talked about it, I felt that she had these expectations: "Well, are you able today?" And if you're able, you sort of have to, 'cause: "Jeez, there's only four days until your next treatment so we better make use of the time". I felt like there was this pressure*. [22-year old man]

### The psychological effects of having genital warts

The psychological, social and sexual effects of having GWs are all interrelated. Some participants had feelings of guilt or anger because they had not protected themselves and others more carefully. Some men were annoyed that they had put off seeking treatment for too long. All participants had developed lower self esteem and a negative body-perception as a result of the disease.

*Sometimes, when you think about it or you notice them [the GWs], you just become so discouraged and sad because...you can't do anything about it. I mean, you just have to wait and there's nothing you can do yourself, is there? And then I get this feeling that I simply can't relate to my own body or even look at it. Then I feel repulsive, you see? *[21-year-old woman]

With time, the participants had learned to live with the disease to some extent, but the lengthy course of treatment, the powerlessness and uncertainty as to when it would end were wearing. The realisation that the disease can remain dormant and return later in life added to the perception of GWs as a serious condition. For the participants who had had symptom-free periods, the psychological impact was severe when the GWs reappeared.

### The social impact of having genital warts

Because of fear of stigmatisation, the participants wanted to control who knew they had GWs. They were worried that others might find them unclean, careless or "of easy virtue" - the latter applied to men as well as women. They were afraid that the disease would be an important factor in shaping others' opinions about them.

*You're afraid of being stigmatised. I remember having heard that somebody had a venereal disease... And that's just what you'll always remember about them, even if you don't even see them anymore. Or if you've heard that about a girl that you might have thought was quite cute, then you would think "wasn't it her who had that thing?" In the same way you think that's probably how others will think about you when you have GWs, that they think "Oh, it's that guy with GWs". That's why I have only told people I'm very close to*. [30-year-old man]

Several participants mentioned that the disease is particularly stigmatising because it is infectious and they were concerned about people's ignorance about the danger of infection. As a consequence, some participants stopped doing sport or other activities that might reveal their disease. Some women felt unattractive because they could not shave intimately as well as they would have liked. Some men were concerned that others might spot the GWs or the ulcerations caused by treatment. The fear of stigmatisation also meant that the participants avoided informing other people that they were attending the venereal diseases clinic for treatment. Many had to take significant time away from their studies or work that was not easy to explain. Most participants had only spoken about their disease to their closest relatives and friends.

### The impact of treatment for genital warts

The participants found that different physicians managed individual treatments and the complete course of treatment for GWs in different ways. Many expressed frustration with a course of treatment that seemed to be inconsistent and experimental. Their expectations of medical solutions were at odds with the fact that there is no 'magic bullet' against this disease. The varying effectiveness of treatment had a considerable impact on the patient's state of mind; improvement or disappearance of the GWs was met with delight, while a recurrence or worsening of the GWs had a correspondingly negative effect.

Several participants expressed a huge need for more knowledge about GWs. Men in particular said they did not like to ask for the information and would have preferred to be offered information about good hygienic practices, how to avoid infection (for themselves and others), the realistic prospects of the course of the disease and its possible psychological and sexual effects.

*These were the kind of things that there was little information about [in the leaflets that the patients were handed at the clinic]. I mean, that you're not alone in feeling that it hurts your soul when you've got this and that you can have problems with your sex life and all such things. There isn't one single leaflet on dangerous diseases, such as AIDS, where it doesn't say something about those psychological things. It's like it [GW] isn't taken that seriously, because after all, it doesn't do that much harm. Even though you actually have a lot of questions like that and there are all these psychological things that hit you*... [23-year-old man]

## Discussion

The participants in this study all expressed a significant reduction in their quality of life as a result of having GWs. The unknown delay to cure, the uncertain perspectives of recovery as well as their inability to control their disease all had a negative psychological effect on them. The stigma associated with the disease affected their self-perception and their social lives. The fact that they had GWs was difficult to ignore because they were constantly reminded, in particular, by the repeated treatments, the disruption of their social lives and the negative effects on their sex and love life (Figure 1). These results are supported by the findings of the few other studies on this subject [3,14,15,32-45].

GWs was considered to be a serious disease, especially by those participants who associated GWs with an increased risk of developing cervical cancer, because both are caused by human papilloma viruses (HPV) [17]. The results of other studies have suggested that this association can greatly influence the perception of illness; conversely, women may regard cervical dysplasia as stigmatising when they are informed about the sexual transmission of HPV [4,32].

In this study, qualitative methodology was used to obtain an in-depth insight into the experiences of patients with GWs. The method of recruitment of the participants may have introduced some selection bias. Patients with a more negative experience of GWs may be more likely to volunteer to participate in a study of this kind. However, the patients with GWs attending the venereal diseases clinic at Bispebjerg Hospital are considered to be representative, in terms of the severity of their symptoms, of Danish patients with GWs in general, in that often they attend the clinic without having previously received treatment elsewhere or they are referred by a general practitioner after just a few attempts at treatment. Finally, the age of our participants may explain why, unlike those in some studies [3,33,35], they did not raise the issues of fertility or pregnancy. The average age of the men and women in this study was 25.8 years and 24.4 years, respectively; in Denmark, the average age at which women have their first child is 29.1 years [51].

We do not think that more focus-group interviews would have produced more qualitative knowledge about the experiences of this age group since the results are consistent with those of previous studies. However, further studies could investigate the effect of GWs on the quality of life of older patients or among ethnic minorities. Since the first publication of the results of this study [52],a follow up study among men having sex with men (MSM) has been published [53] and a study of the possible long-term effects of GW is currently in press (Ugeskrift for Læger).

## Conclusions

This study contributes to our knowledge about patient perspectives on HPV-related diseases and it suggests that GWs affect men as much as women, psychologically, socially and sexually. Men and women in this study described experiences that were related to cultural perceptions of venereal diseases and the respective identities (and sexuality) of the sexes. Cultural notions of the male identity, sexual desire and performance were at odds with the feelings of vulnerability and lack of desire expressed by these men. Our results suggest that there is a considerable need for more patient information about the disease and improved doctor-patient communication. Men, in particular, seemed to experience psychological barriers to seeking information and counselling. It is clear that although GWs are not a life-threatening disease from a medical practitioner's point of view, they have wide-ranging effects on the patient's quality of life, and these need to be taken into consideration.

The development of vaccines against HPV is a milestone in the prevention of GW, as well as cervical lesions and cancer. The quadrivalent HPV vaccine that has now been added to the childhood vaccination programme for girls in Denmark could prevent 90% of genital warts. Our results suggest that HPV vaccination could contribute substantially to reducing the largely unacknowledged psychological and social burden associated with genital warts, in men as well as in women.

## Competing interests

GLM has received a research grant for this study from Sanofi Pasteur MSD. The study was conceived and the protocol was developed independently of this funding. HKL: None

## Authors' contributions

GLM conceived the study and its design, carried out the data collection, analysed and interpreted the data and drafted the manuscript. HKL participated in the inclusion of patients, drafted the medical parts of the manuscript and revised the whole manuscript critically for important intellectual content. Both authors have read and approved the final manuscript.

## Authors' informations

GLM has a MA in social anthropology and is specialised in medical anthropology. She currently works as a research consultant at AnthroConsult specialised in qualitative patient studies.

## Pre-publication history

The pre-publication history for this paper can be accessed here:

http://www.biomedcentral.com/1471-2458/10/113/prepub
